# Dynamics and spike trains statistics in conductance-based Integrate-and-Fire neural networks with chemical and electric synapses

**DOI:** 10.1186/1471-2202-14-S1-P58

**Published:** 2013-07-08

**Authors:** Rodrigo Cofre, Bruno Cessac

**Affiliations:** 1 NeuroMathComp team (INRIA, ENS Paris, UNSA LJAD), Sophia Antipolis, France

## Introduction

Communication between neurons involves chemical and electric synapses. Electric synapses transmission is mediated by gap junctions providing direct communication between cells, allowing faster communication than chemical synapses. Electrical coupling between cells can be found in many parts of the nervous system.

At the network level, electric synapses have several prominent effects such as neural synchronization, and generation of neural rhythms. Additionally, they are responsible for sharp peaks in the auto-correlation of ganglion cells in the retina. Dealing with spike population coding, interactions between neurons highly constrain the collective spike response of a neural assembly to stimuli. Thus, unveiling the respective effect of chemical and electric synapses on spike responses is a mandatory step towards a better understanding of spike coding.

Can we have a reasonable idea of what is the spike train statistics and how it depends on stimulus and connectivity studying a neural network model considering chemical and electrical synapses? This work answers this question.

## Methods and results

In [1] we have analyzed mathematically the collective effects of chemical and electric synapses in a conductance-based Integrate-and-Fire neural network, where conductances depend on spike history [2]. We have also analyzed spike train statistics and show that it is described by a non-stationary Gibbs distribution whose potential can be approximated with an explicit formula, when the noise is weak. This result is compared to maximum entropy models currently used in the literature of spike train analysis. The Gibbs potential includes existing models for spike trains statistics analysis such as maximum entropy models or Generalized Linear Models (GLM). The potential has an infinite range (infinite memory), although Markovian approximation can be proposed, replacing the exact potential by a potential with a finite range. The Gibbs distribution obtained in our model is quite more complex than Ising model or Generalized Linear Models used in retinal spike train analysis [3]. Especially, it involves spatio-temporal spike patterns and is non stationary. We identified the role of gap junctions on history dependence and in the spike train statistics. We also discuss the different types of correlations: those induced by a shared stimulus and those induced by chemical and electric interactions between neurons.

## Conclusion

This work suggest that electric synapses could have a strong influence in spike train statistics of biological neural networks, especially in the retina where gap junctions connections between several cells-type (e.g. amacrine and ganglion cells or amacrine-bipolar) are ubiquitous. The main observation is that considering gap junctions the probability of spike patterns does not factorize as a product of marginal, per-neuron, distributions. Therefore there is absolutely now way to defend that neurons in this model act as independent sources. Additionally, correlations mainly result from the chemical and electrical interactions (correlations persist even if there is no external current / stimulus), thus gap junctions play an important role in correlating the network even in absence of stimulus. This work provides a firm theoretical ground for recent studies attempting to describe experimental rasters in the retina as well as in the parietal cat cortex by Gibbs distributions using the maximum entropy principle.
